# Research progress on the mechanism of chondrocyte ferroptosis in osteoarthritis

**DOI:** 10.3389/fimmu.2026.1790918

**Published:** 2026-03-27

**Authors:** Ling Zhou, Shaohua Ju, Kun Wang, Zhibin Fu, Ruicheng Wu, Benxiang He, Yushi Hu

**Affiliations:** 1 Orthopedics and Arthrology Department, Affiliated Sports Hospital of Chengdu Sport University, Chengdu, China; 2 Pharmacy Department, Chengdu Sports University Affiliated Sports Hospital, Chengdu, China; 3 Sports Medicine and Health College, Chengdu Sports University, Chengdu, China; 4 School of Clinical Medicine, Chengdu Medical College, Chengdu, China; 5 Sichuan Academy of Traditional Chinese Medicine, Chengdu, China

**Keywords:** chondrocytes, ferroptosis, lipid peroxidation, mechanism of action, osteoarthritis, research progress, targeted therapy

## Abstract

Osteoarthritis (OA) is a chronic, progressive joint disease characterized by degenerative changes in articular cartilage, subchondral bone sclerosis, accompanied by synovitis and chondrocyte apoptosis. With the aging of society, it has become one of the major factors endangering the mobility of middle-aged and elderly people worldwide. The pathogenesis of OA remains unclear, and current treatments can only control symptoms without effectively repairing damaged cartilage. Ferroptosis, a novel form of programmed cell death proposed and confirmed in 2012, is characterized by iron-dependent accumulation of lipid peroxides, mitochondria-specific damage, and imbalance in antioxidant defense capacity. In recent years, it has been proven to be involved in regulating the process of cartilage degradation in OA. This review mainly focuses on the key biological processes of chondrocyte ferroptosis, elaborating on the interactions between iron homeostasis disorders, oxidative stress and inflammation, as well as the research progress on chondrocyte ferroptosis induced by dysregulation of important signaling pathways in the pathological environment of OA. In summary, it systematically analyzes the relationship between ferroptosis and other forms of programmed cell death, summarizes the research progress of OA therapeutic strategies targeting chondrocyte ferroptosis, and puts forward the existing contradictions and development trends in current research, aiming to provide new clues and directions for exploring the mechanism of OA occurrence and development and clinical personalized treatment.

## 1 Introduction

As the most common musculoskeletal disorder worldwide, epidemiological surveys have shown that the incidence of OA exceeds 50% among people over 60 years old globally. Moreover, the incidence of OA continues to rise due to the increasing obesity rate and the extension of human average lifespan (PubMed). OA is mainly manifested by the gradual destruction of articular cartilage, decreased number of chondrocytes, synovitis, subchondral bone remodeling, and formation of proliferative osteophytes, which eventually develop into joint pain, stiffness, dysfunction, and even disability, imposing a huge burden on families and the social medical and health system (1, 2).

As the weight-bearing pad of joints, the normal physiological activities of articular cartilage are highly dependent on the stable state of chondrocytes. Chondrocytes are the only cells in cartilage that synthesize extracellular matrix (ECM) such as type II collagen (COL2A1) and proteoglycans, regulate the expression of degrading enzymes such as matrix metalloproteinases (MMPs), and maintain the dynamic balance between ECM synthesis and degradation. During the development of OA, chondrocyte apoptosis is one of the important initiating mechanisms of cartilage destruction (2, 3). Traditional views hold that apoptosis and autophagy are the main ways to regulate chondrocyte death, but recent studies have found that novel forms of programmed cell death such as pyroptosis, necroptosis, and ferroptosis also play important roles in OA progression, which cannot be fully explained by traditional cell death mechanisms.

Ferroptosis was first named by Dixon et al. in 2012, which is a novel iron-dependent programmed cell death process different from apoptosis, necrosis, and autophagy. The main manifestations of ferroptosis include excessive accumulation of intracellular Fe²^+^, abnormal lipid peroxidation of membrane polyunsaturated fatty acids (PUFAs) leading to the production of toxic substances such as malondialdehyde (MDA); abnormalities in the glutathione (GSH)-glutathione peroxidase 4 (GPX4) antioxidant system; and specific changes in mitochondrial ultrastructure: reduction or disappearance of mitochondrial cristae, outer membrane rupture, and increased membrane density (PubMed).

Different from other forms of cell death, ferroptosis has its own characteristics, which is mainly the result of simultaneous dysregulation of three pathways: iron metabolism, lipid metabolism, and antioxidant system. Abnormal iron metabolism leading to excessive iron ions in cells is a prerequisite for ferroptosis (4, 5). A large amount of Fe²^+^ can promote the production of reactive oxygen species (ROS) through the Fenton reaction, initiating the lipid peroxidation chain reaction; lipid metabolism disorders provide substrates for lipid peroxidation, such as long-chain acyl-CoA synthetase 4 (ACSL4) involved in the synthesis and metabolism of unsaturated fatty acids; GPX4 is the main enzyme that scavenges lipid peroxides, and the decrease in GPX4 activity or expression is the central link of ferroptosis (PubMed).

Since ferroptosis was proposed, more and more studies have focused on its contribution to degenerative diseases. Relevant studies after 2018 have shown that abnormal expression of ferroptosis-related markers can be detected in the cartilage tissue of OA patients: downregulated expression of GPX4, while high expression of ACSL4 and transferrin receptor 1 (TfR1), increased Fe²^+^ and MDA, and decreased GSH, suggesting that chondrocyte ferroptosis may play a role in the pathogenesis of OA. Moreover, recent studies demonstrate that mechanical confinement can directly trigger ferroptosis through nuclear deformation, Drp1-dependent mitochondrial fragmentation, and cPLA2-mediated lipid peroxidation, highlighting a mechanistic link between mechanical stress and chondrocyte ferroptosis in OA (6).

Subsequent cellular and animal model experiments have also confirmed this hypothesis: treatment of chondrocytes with ferroptosis inducers (Erastin, RSL3) can significantly accelerate their apoptosis and ECM degradation (7, 8), while ferroptosis inhibitors (Ferrostatin-1, Fer-1) can effectively reverse this situation; in OA animal models, targeted intervention of chondrocyte ferroptosis can significantly alleviate the degree of cartilage degeneration, reduce the expression of inflammatory factors, and promote the recovery of joint function. Although increasing studies have reported the involvement of ferroptosis in osteoarthritis, current evidence remains fragmented and sometimes inconsistent, particularly regarding its temporal role and regulatory hierarchy in OA progression. Moreover, few reviews have systematically integrated iron homeostasis, lipid metabolic remodeling, antioxidant system dysfunction, and mitochondrial impairment into a unified mechanistic framework, nor have they comprehensively summarized recent translational efforts targeting ferroptosis in OA. Therefore, this review aims to provide a structured and integrative overview of chondrocyte ferroptosis in OA by organizing its core biological processes into a coherent regulatory network, discussing existing controversies, and summarizing emerging therapeutic strategies. By linking mechanistic insights with translational implications, we seek to clarify the role of ferroptosis within the complex pathological landscape of OA.

## 2 Core biological mechanisms of chondrocyte ferroptosis

### 2.1 Iron metabolism imbalance and iron overload

**Figure 1:**
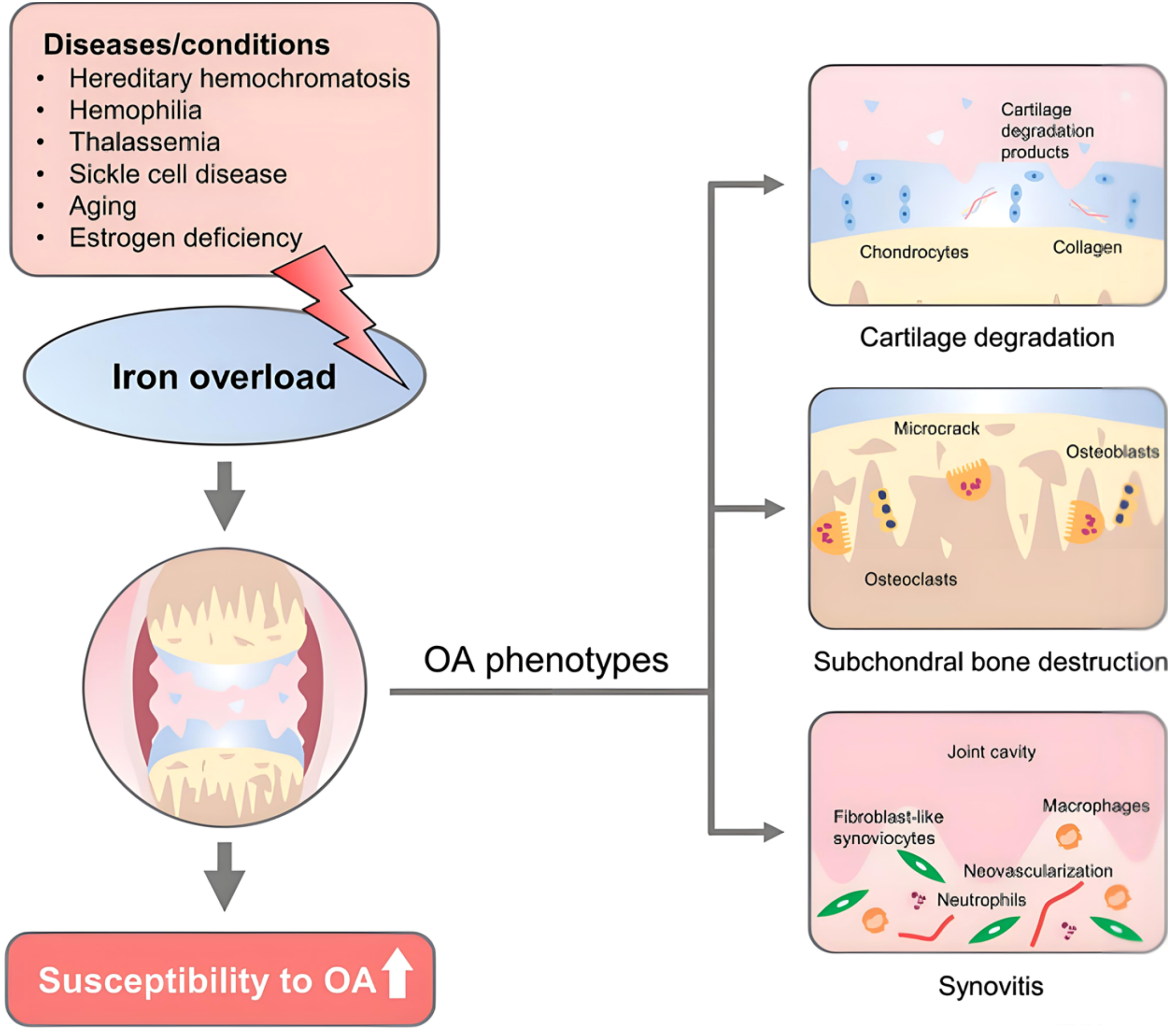
Etiology of iron metabolism and iron overload and osteoarthritis phenotype.

Iron, as an essential trace element for cell physiological metabolism, plays an important role in heme synthesis, electron transfer, and enzyme catalysis; however, excessive iron can cause oxidative stress damage through the Fenton reaction. Under normal circumstances, iron metabolism in cells is regulated by a circulatory system of “absorption-storage-efflux” to achieve a relatively stable state. In OA, this balance of chondrocyte iron metabolism is disrupted, leading to iron accumulation and increased sensitivity to ferroptosis, as shown in **Figure 1**.

#### 2.1.1 Enhanced iron uptake

Cellular iron uptake is mainly through TfR1 transferrin (Tf)-Fe³^+^ endocytosis. Inflammatory factors (IL-1β, TNF-α) in the OA pathological microenvironment can increase the expression of TfR1 by activating the NF-κB signaling pathway, thereby promoting iron uptake by chondrocytes. Studies have shown that treatment with IL-1β significantly increases the mRNA and protein levels of TfR1 in human chondrocytes, and silencing of TfR1 promotes Fe²^+^ accumulation, induces ferroptosis, and alleviates ECM degradation (9, 10); in addition, OA cartilage is accompanied by decreased expression of ferroportin 1 (FPN1), the only extracellular iron exporter, resulting in more significant intracellular iron accumulation (PubMed).

#### 2.1.2 Disordered iron storage

Ferritin is the main protein for intracellular iron storage, a complex composed of heavy chain (Ferritin-H) and light chain (Ferritin-L), which can convert free Fe²^+^ into Fe³^+^ and store it in the protein shell to prevent oxidative damage; the expression of Ferritin-H in the cartilage tissue of OA patients is significantly downregulated, leading to decreased iron storage and increased free Fe²^+^ (PubMed). scRNA-seq analysis shows that there is a subpopulation of chondrocytes with higher ferroptosis activity in OA cartilage tissue, and the expression of Ferritin-H in this subpopulation is much lower than that in normal chondrocytes (11, 12), indicating that iron storage dysfunction is one of the reasons why chondrocytes are susceptible to ferroptosis.

#### 2.1.3 Abnormal regulation of iron metabolism-related genes

In addition to iron transporters and storage proteins, many genes are involved in the regulation of chondrocyte iron metabolism. Studies have shown that transcription factor specificity protein 1 (SP1) can enhance ACSL4 transcription by directly binding to the promoter region of ACSL4. ACSL4 itself is involved in lipid metabolism and can aggravate iron overload by upregulating iron transporters; P53 is a classic tumor suppressor gene. After abnormal activation in OA chondrocytes, it can reduce GSH levels by inhibiting the expression of SLC7A11 and increase iron intake by upregulating the expression of TfR1 (13, 14), thereby bidirectionally regulating the process of ferroptosis.

### 2.2 Lipid peroxidation and cell membrane damage

**Figure 2:**
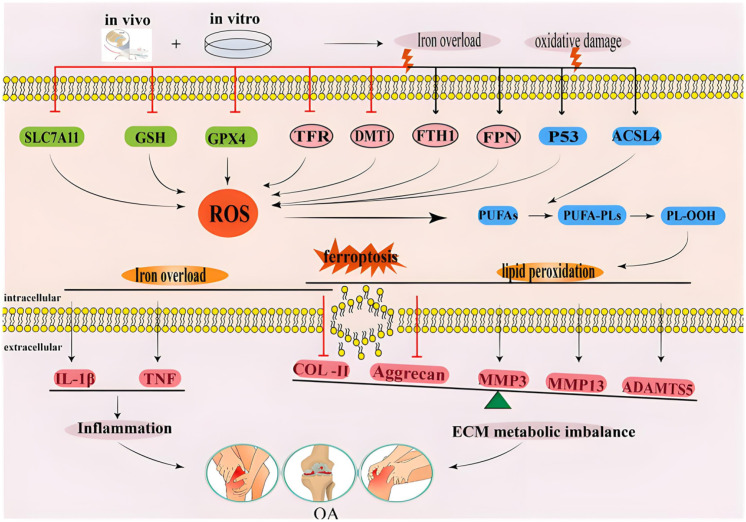
Regulatory mechanism of lipid peroxidation and cell membrane damage in osteoarthritis.

The key biochemical process of ferroptosis is lipid peroxidation. Under the action of ROS, unsaturated fatty acids on the cell membrane undergo chain oxidation reactions to produce a large number of lipid peroxidation products (MDA, 4-HNE), resulting in the destruction of cell membrane integrity and cell death. Chondrocyte membranes are rich in unsaturated fatty acids, while cartilage tissue has weak antioxidant capacity and is susceptible to lipid peroxidation damage, as shown in **Figure 2**.

#### 2.2.1 Initiation and amplification of lipid peroxidation

The Fenton reaction caused by iron overload is the main pathway leading to lipid peroxidation: excessive Fe²^+^ reacts with H_2_O_2_ to produce ▪OH, which can directly attack the double bonds on unsaturated fatty acids to generate lipid free radicals (L▪); L▪ reacts with O_2_ to produce lipid peroxyl radicals (LOO▪), which attack other unsaturated fatty acid molecules again, triggering a lipid peroxidation chain reaction and achieving amplified damage (PubMed) (15, 16). The increased level of oxidative stress in the OA pathological microenvironment further aggravates this process. Members of the NADPH oxidase (NOX) family (especially NOX1) are activated to produce a large amount of ROS, providing the prerequisite for lipid peroxidation.

#### 2.2.2 Regulatory role of key lipid metabolic enzymes

Abnormal expression of lipid metabolism-related enzymes is also involved in regulating the lipid peroxidation reaction in chondrocyte ferroptosis. ACSL4 is the main enzyme involved in the synthesis of long-chain unsaturated fatty acids, which can convert unsaturated fatty acids such as arachidonic acid into acyl-CoA and then participate in lipid peroxidation. Therefore, high expression of ACSL4 is also one of the manifestations of ferroptosis in OA chondrocytes (PubMed). In summary, IL-1β transcriptionally activates the expression of ACSL4 via SP1; silencing ACSL4 can significantly reduce the degree of lipid peroxidation and ferroptosis in chondrocytes, promote the expression of COL2A1, and inhibit the secretion of MMP13.

In addition, members of the LOX family (such as ALOX5) also play a role in the process of chondrocyte ferroptosis. Bioinformatics analysis found that in OA cartilage tissue, the expression of ALOX5 is significantly increased, which is inversely proportional to the ferroptosis marker molecule GPX4 (17, 18). Its overexpression can promote the lipid peroxidation reaction by catalyzing the oxidation of unsaturated fatty acids. Inhibiting the activity of ALOX5 can reduce chondrocyte ferroptosis (PubMed).

#### 2.2.3 Dysfunction of antioxidant system

Under normal physiological conditions, cells scavenge ROS and lipid peroxides through various antioxidant systems to maintain redox balance. Among them, the GSH-GPX4 system is the core antioxidant pathway inhibiting ferroptosis: GSH, as a reducing coenzyme, provides reducing equivalents for GPX4, and GPX4 reduces lipid peroxides to non-toxic lipid alcohols, blocking the lipid peroxidation chain reaction.

The GSH-GPX4 function is dysfunctional in OA chondrocytes: (1) Inflammatory factors and oxidative stress (OS) can inhibit the expression of SLC7A11, a core subunit of the cystine/glutamate antiporter (System Xc^-^). Its dysfunction leads to insufficient cystine intake, which is an essential precursor for GSH synthesis (19, 20), ultimately resulting in decreased GSH synthesis; at the same time, oxidative stress induced by NOX1 can directly inhibit the Nrf2/HO-1 pathway. Nrf2 is an important antioxidant response element, and GPX4 and HO-1 are its downstream molecules. When the Nrf2/HO-1 pathway is inhibited, the level and enzyme activity of GPX4 decrease, affecting the degradation of lipid peroxides.

Beyond the classical GSH–GPX4 axis, emerging evidence suggests that ferroptosis can also be modulated through GPX4-independent antioxidant systems. Ferroptosis suppressor protein 1 (FSP1), originally identified as a CoQ10 oxidoreductase, has recently attracted attention in cartilage biology. In TMJ osteoarthritis models, FSP1-expressing cells in the superficial zone were shown to be essential for cartilage maintenance, and disruption of this population resulted in severe cartilage degeneration (21). Moreover, in inflammatory chondrocyte models, FSP1 expression was reduced and functionally linked to increased oxidative stress and ferroptosis, as silencing of FSP1 abolished the protective effects of exosomal SNHG7 (22). Although mechanistic details remain limited, these findings suggest that FSP1 may participate in the impaired antioxidant defense network in OA and represent a complementary regulatory layer beyond GPX4.

### 2.3 Mitochondrial dysfunction and ferroptosis

**Figure 3:**
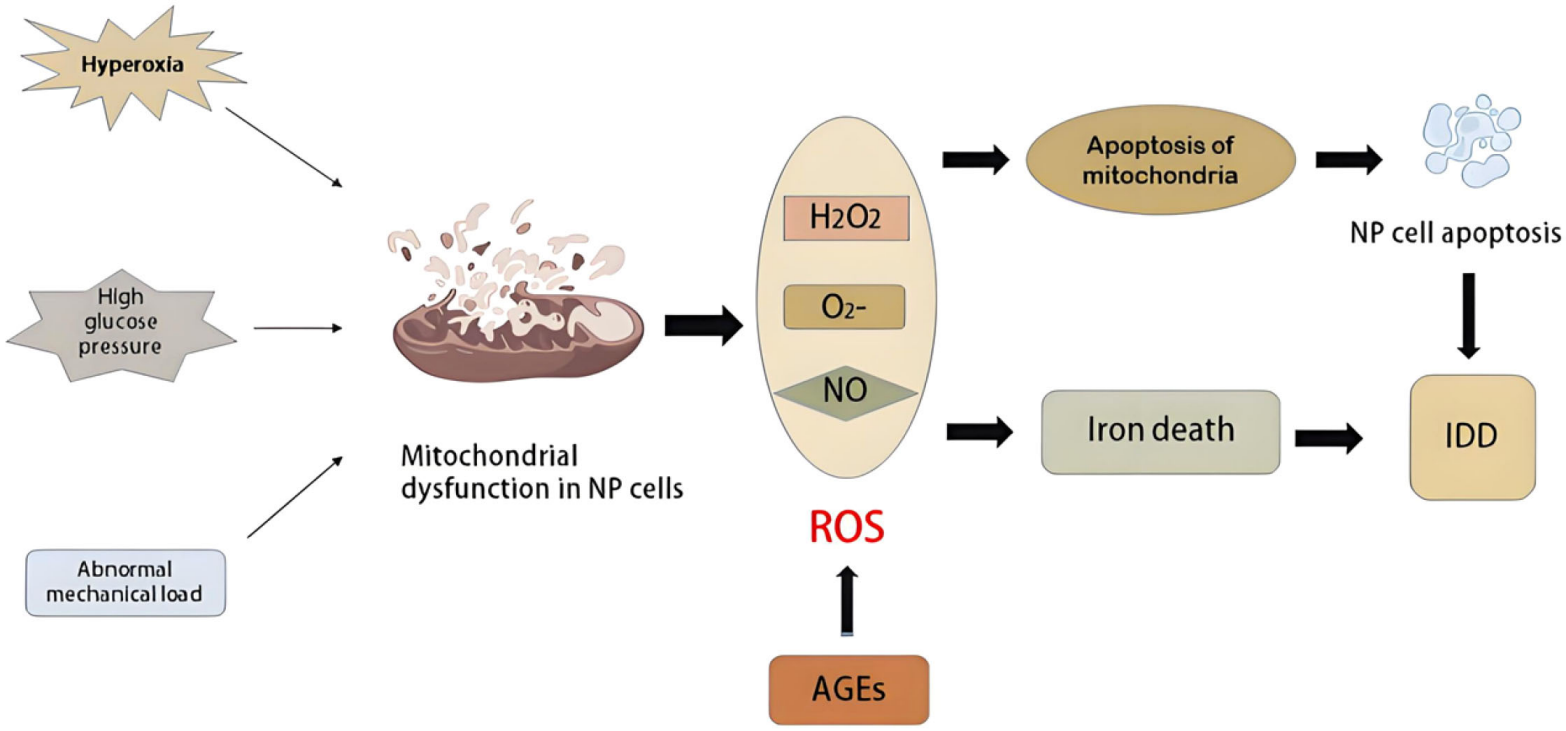
Mitochondrial dysfunction and ferroptosis in osteoarthritis.

Mitochondria are the energy factories of cells and the main site of ROS production. In ferroptosis, they can be regarded as both the target organ of ferroptosis and the main source of ferroptosis-related ROS. Like other cells, chondrocytes also show classic mitochondrial structural abnormalities during ferroptosis (23, 24), but due to the metabolic characteristics of chondrocytes themselves (hypoxia, mainly glycolysis), their regulation of mitochondrial dysfunction in ferroptosis has certain specificity, as shown in **Figure 3**.

#### 2.3.1 Specific changes in mitochondrial structure and function

Transmission electron microscopy shows that after the action of ferroptosis inducers, chondrocyte mitochondria shrink in volume, mitochondrial cristae decrease or disappear, the outer membrane ruptures, and the mitochondrial membrane potential decreases significantly. Moreover, the above changes occur before cell membrane damage, indicating that mitochondrial damage is an early event in the process of chondrocyte ferroptosis; in terms of function, abnormal mitochondrial respiratory chain function is also one of the reasons for increased ROS production: iron overload can lead to mitochondrial iron accumulation, affect the stability of respiratory chain complexes, cause obstruction of the electron transport chain, electron leakage and combination with oxygen to produce a large amount of mitochondrial ROS (mtROS) (25, 26), which aggravates the lipid peroxidation reaction and promotes ferroptosis.

#### 2.3.2 Regulatory role of mitophagy

Mitophagy refers to the removal of damaged mitochondria through autophagic pathways, which can reduce mtROS production and iron deposition, and play a negative regulatory role in chondrocyte ferroptosis. Studies have shown that Sestrin2 (Sesn2) is one of the important stress response proteins. The expression of Sesn2 in chondrocytes is significantly reduced in OA. Overexpression of Sesn2 can induce mitophagy by transcriptionally increasing the expression of BNIP3, reduce mitochondrial iron accumulation and mtROS production, thereby increasing the activity of the Nrf2/GPX4 pathway and inhibiting ferroptosis (27, 28). *In vivo* experiments found that intra-articular injection of adeno-associated virus overexpressing Sesn2 (AAV-Sesn2) can significantly alleviate cartilage degeneration and reduce the expression of ferroptosis markers in DMM mice, indicating that mitophagy can inhibit chondrocyte ferroptosis by regulating mitochondrial function.

## 3 Regulatory network of chondrocyte ferroptosis in osteoarthritis

### 3.1 Iron metabolism-related regulatory pathways

The P53/SLC7A11/GPX4 pathway is a classic pathway regulating cellular ferroptosis and plays a key role in OA chondrocyte ferroptosis. P53 is a transcription factor. When activated by oxidative stress and inflammatory signals in the pathological microenvironment of OA, it can directly bind to the inhibitory elements on the SLC7A11 promoter, block the transcriptional expression of the SLC7A11 gene, reduce cystine uptake in chondrocytes, insufficient GSH production, further downregulate GPX4 activity, and induce lipid peroxidation and ferroptosis.

This study detected through clinical specimens that P53 is significantly upregulated in the cartilage of OA patients, while the expressions of SLC7A11 and GPX4 are significantly downregulated, and the expressions of the three show a pairwise negative correlation; *in vitro* experiments confirmed that silencing P53 can significantly improve the expressions of SLC7A11 and GPX4, reduce the production of Fe²^+^ and MDA, alleviate chondrocyte ferroptosis and ECM damage, while overexpression of P53 aggravates the above pathological processes (PubMed) (29, 30). Some scholars have conducted therapeutic research on metformin and found that metformin can inhibit the activity of P53 by directly binding to the P53 protein, increase the expressions of SLC7A11 and GPX4, thereby correcting iron metabolism disorders and lipid peroxidation, and reducing cartilage damage in OA model rats.

The Nrf2/HO-1 pathway is an important antioxidant defense pathway in the body, which can maintain intracellular redox balance by regulating the expression of downstream antioxidant genes and play an important inhibitory role in chondrocyte ferroptosis. In the inactive state, Nrf2 binds to Keap1 and localizes in the cytoplasm. When activated by oxidative stress or drugs, it dissociates from the Keap1 complex, translocates to the nucleus, binds to the antioxidant response element (ARE), and initiates the transcriptional expression of GPX4, HO-1, and SOD genes (31, 32), increasing cellular antioxidant capacity.

During the occurrence and development of OA, NOX1 oxidative stress can weaken the Nrf2/HO-1 signaling pathway by inhibiting the nuclear translocation of Nrf2, reduce the expressions of GPX4 and HO-1, weaken the antioxidant function of chondrocytes, and make them more sensitive to ferroptosis. Activation of Nrf2 significantly reduces chondrocyte ferroptosis. In the IL-1β-induced OA cell model, drugs activating Nrf2 (sulforaphane) can significantly increase the expressions of GPX4 and HO-1, reduce the levels of ROS and MDA, and inhibit ferroptosis and ECM degradation (33, 34); *in vivo* experiments have proved that activation of Nrf2 can significantly alleviate cartilage degeneration and synovitis symptoms in DMM mouse models, indicating that Nrf2/HO-1 is an important protective pathway regulating chondrocyte ferroptosis.

**Figure 4:**
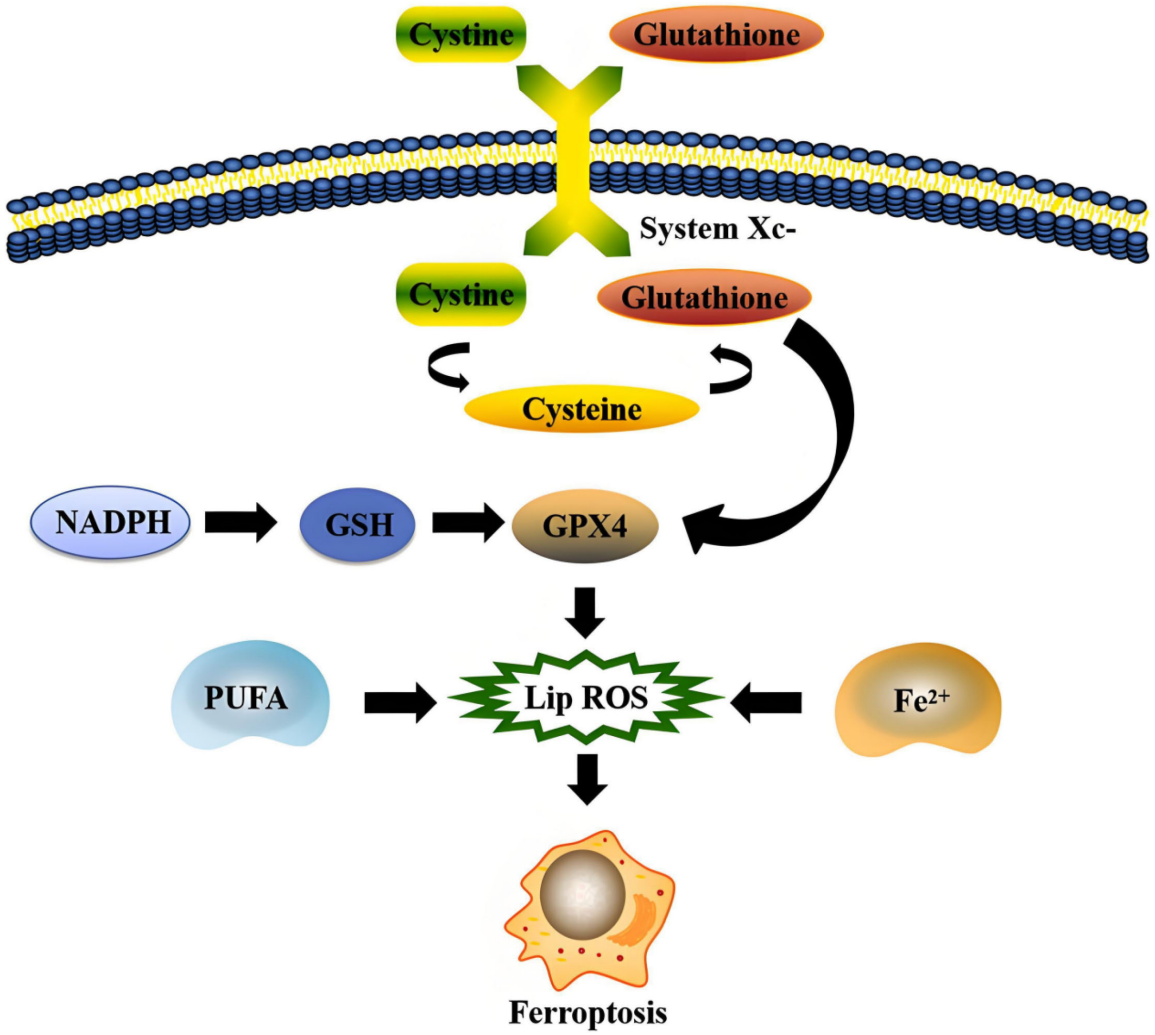
Iron metabolism-related regulatory pathways in osteoarthritis.

The yellow transmembrane transport complex on the cell membrane shown in **Figure 4** is the key node connecting the above pathways with the core process of ferroptosis. As a cystine/glutamate antiporter, it is responsible for transporting extracellular cystine into the cell and converting it into cysteine, which is a necessary prerequisite for the synthesis of glutathione (GSH). GSH needs to provide reducing equivalents for GPX4 with the energy supply of NADPH to scavenge lipid reactive oxygen species (Lip ROS) produced by the metabolism of Fe²^+^ and polyunsaturated fatty acids (PUFAs). Combined with the aforementioned P53 pathway, activated P53 in the OA pathological microenvironment can directly inhibit the transcription of SLC7A11 (35, 36), and inflammatory factors can also disrupt the complex stability of this transporter, reducing its cystine uptake capacity; this change leads to insufficient intracellular cysteine, limited GSH synthesis, thereby weakening the ability of GPX4 to scavenge Lip ROS, and ultimately promoting the massive accumulation of Lip ROS, triggering chondrocyte ferroptosis, which is completely consistent with the occurrence process of ferroptosis after the dysfunction of System Xc^-^ shown in the figure.

### 3.2 Cross-regulation of oxidative stress and inflammatory pathways

#### 3.2.1 NOX1-ROS-NF-κB Axis

**Figure 5:**
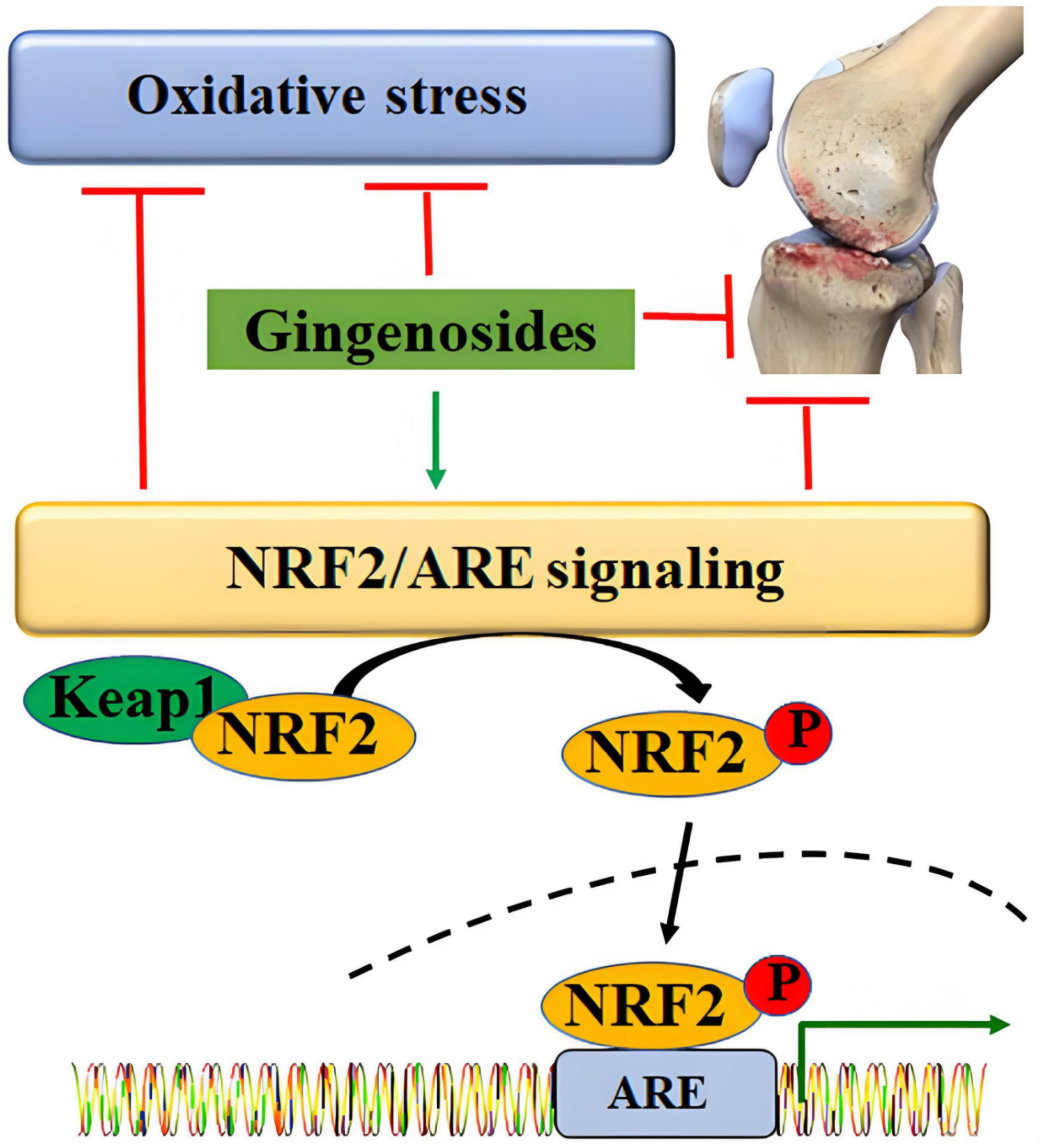
Cross-regulation of oxidative stress and inflammatory pathways in osteoarthritis.

The NOX1-ROS-NF-κB axis is a key regulatory axis linking oxidative stress, inflammatory response, and chondrocyte ferroptosis. NOX1 is a subtype of the NOX family, highly expressed in OA chondrocytes. Its activation can catalyze the oxidation of NADPH to produce a large amount of ROS, which can directly initiate lipid peroxidation or activate the NF-κB pathway, triggering an inflammatory cascade reaction, as shown in **Figure 5**.

NF-κB is a classic inflammatory transcription factor. Its activation can promote chondrocyte ferroptosis through two pathways: one is to upregulate pro-inflammatory factors such as IL-1β and TNF-α, which can activate NOX1 to produce ROS, forming a positive feedback reaction of oxidative stress-inflammation (37, 38); the other is to directly upregulate the expression of TfR1 and ACSL4 to promote iron intake and the synthesis of lipid peroxidation substrates, and on the other hand, to weaken the antioxidant effect by silencing the expression of SLC7A11 and GPX4. Studies have shown that silencing NOX1 can significantly reduce the ROS content in OA chondrocytes, inhibit the activation of the NF-κB signaling pathway, upregulate the activity of the Nrf2/HO-1 signaling pathway, thereby reducing the occurrence of ferroptosis, and restore the downregulated expression of COL2A1 and the upregulated expression of MMP13.

#### 3.2.2 ROS-NLRP3 inflammasome-mediated synergistic effect of pyroptosis and ferroptosis

Recent reports suggest that ferroptosis and pyroptosis promote each other in the pathological process of OA, participating in chondrocyte apoptosis and the occurrence and development of arthritis, and the ROS-NLRP3 inflammasome is an important intersection. The NLRP3 inflammasome is a key signaling protein for pyroptosis, composed of NLRP3, ASC, and Caspase-1, which is activated under the stimulation of damage-associated molecular patterns (DAMPs) such as ROS and ATP.

ROS produced by iron overload in OA chondrocytes can directly activate the NLRP3 inflammasome. Activated Caspase-1 cleaves GSDMD to form its N-terminal fragment, which forms pores on the cell membrane to cause pyroptosis and produce pro-inflammatory factors such as IL-1β and IL-18 (39, 40); the above pro-inflammatory factors further activate the NF-κB signaling pathway by binding to the corresponding receptors on chondrocytes, upregulate the expression of NOX1 and TfR1, aggravate ROS production and iron overload, and promote ferroptosis; on the other hand, mitochondrial DNA and high mobility group box 1 (HMGB1) produced by ferroptosis can act as DAMPs to activate the NLRP3 inflammasome to trigger pyroptosis again, and the two can amplify each other, leading to further deterioration of the condition. Studies have found that blocking the NLRP3 inflammasome can reduce both pyroptosis and the expression of ferroptosis-related indicators, significantly alleviate cartilage degeneration and inflammatory response in OA model mice, suggesting that targeting this synergistic pathway may be a new strategy for the treatment of OA.

### 3.3 Regulatory roles of non-coding RNAs and transcription factors

#### 3.3.1 Transcription factor regulation

In addition to the aforementioned P53, Nrf2, NF-κB, and SP1, many other transcription factors are involved in the precise regulation of chondrocyte ferroptosis. YY1 and SREBF1 are recently discovered ferroptosis-related transcription factors through single-cell transcriptome analysis (41, 42). In the ferroptosis-active chondrocyte subpopulation (HOMC) highly expressed in OA cartilage tissue, YY1 and SREBF1 form a specific regulatory network. MMPs directly bind to the ACSL4 promoter region to promote its transcriptional expression, accelerating chondrocyte ferroptosis and ECM degradation.

In addition, TRPM7 is a transient receptor potential channel enzyme. After abnormal activation in OA chondrocytes, it can mediate calcium ion influx, activate the downstream CaN-NFAT signaling pathway, upregulate the expression of TfR1 and ACSL4, promote iron overload and lipid peroxidation. Inhibiting TRPM7 can significantly reduce the occurrence of chondrocyte ferroptosis and improve the joint function of OA model rats (PubMed) (43, 44). These studies suggest that the transcription factor network can regulate the expression of ferroptosis-related genes at multiple targets and play an important role in the pathological process of OA.

#### 3.3.2 Non-coding RNA regulation

Non-coding RNAs (ncRNAs) are a class of RNA molecules involved in the regulation of gene expression. In recent years, studies on the involvement of ncRNAs in chondrocyte ferroptosis have gradually increased. miRNAs regulate the expression of target genes by targeting the 3’ untranslated region of ferroptosis-related genes. For example, miR-146a is highly expressed in OA cartilage tissue and can directly target SLC7A11 to inhibit its expression, upregulate GSH, and inhibit chondrocyte ferroptosis; downregulated expression of miR-214 can activate the antioxidant pathway by relieving the inhibition of Nrf2, thereby inhibiting ferroptosis (PubMed).

lncRNAs and circRNAs mainly regulate miRNA functions in the form of ceRNAs to indirectly regulate the ferroptosis process. For example, lncRNA MALAT1 is highly expressed in OA chondrocytes and can act as a ceRNA to capture miR-124 (45, 46), antagonizing the inhibitory effect of miR-124 on TfR1, and participating in iron absorption and ferroptosis; circRNA HIPK3 can upregulate the expression of GPX4 by adsorbing miR-34a, inhibiting chondrocyte ferroptosis (PubMed). Bioinformatics analysis shows that there are multiple ferroptosis-related ncRNA regulatory networks in OA, which may jointly regulate iron metabolism and antioxidant pathways, revealing the factors affecting the sensitivity of chondrocyte ferroptosis, and can be used as new molecular targets for OA for in-depth research.

## 4 Interaction between chondrocyte ferroptosis and the pathological microenvironment of osteoarthritis

### 4.1 Mechanical stress and chondrocyte ferroptosis

**Figure 6:**
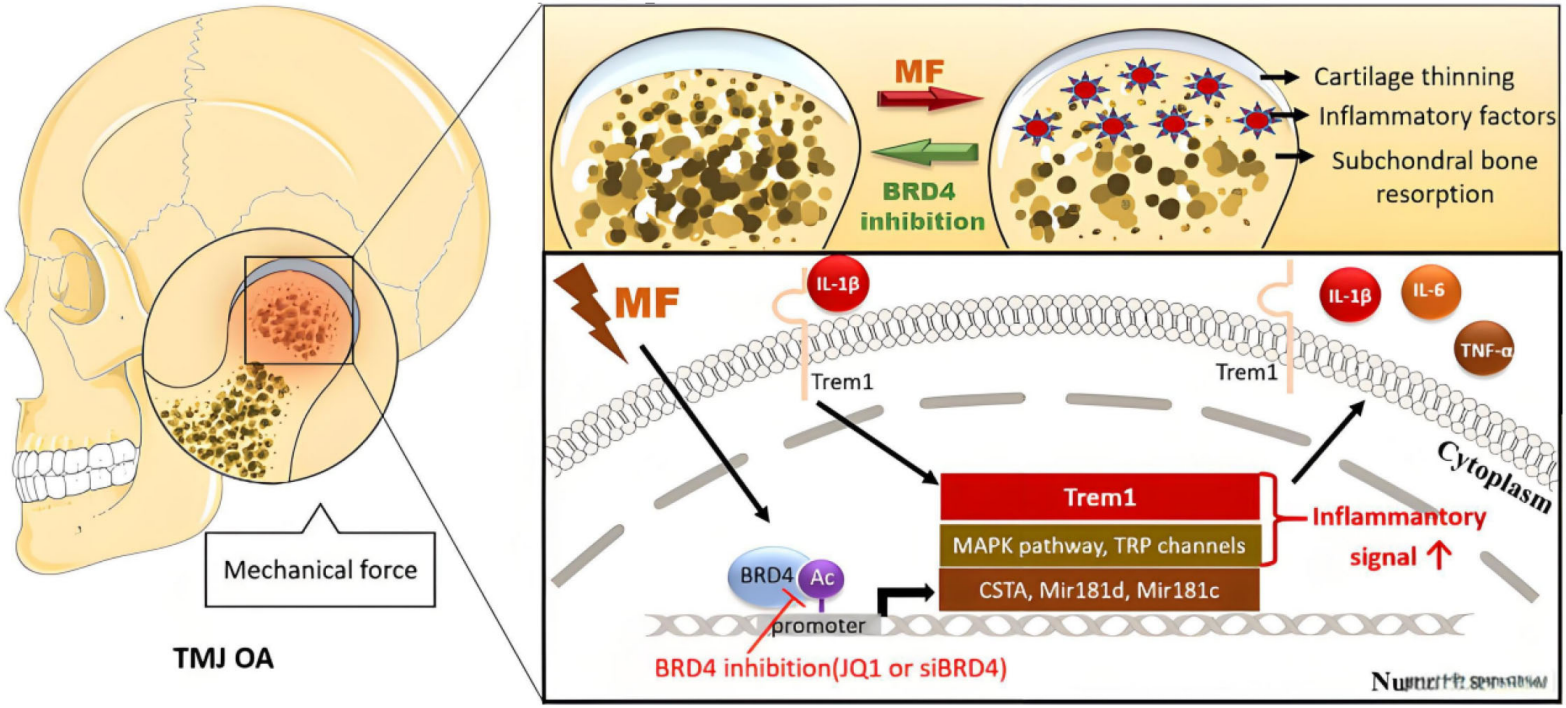
Chondrocyte ferroptosis and mechanical stress.

Risk factors for OA include mechanical stress such as excessive weight-bearing and joint instability. These abnormal mechanical stresses can participate in OA development by regulating chondrocyte ferroptosis. Within the physiological range, mechanical stress can maintain the stability of chondrocytes; while under pathological conditions, prolonged mechanical pressure stimulation and shear force can induce chondrocyte ferroptosis through different pathways, as shown in **Figure 6**.

*In vitro* studies have found that continuous mechanical compression (1MPa, 24h) can significantly increase the expression levels of TfR1 and NOX1 in chondrocytes, reduce the expression of SLC7A11 and GPX4, increase Fe²^+^ and ROS, induce ferroptosis and destroy ECM; mechanical intervention within the physiological range can improve the above conditions and protect chondrocytes (PubMed) (47, 48). The possible mechanism is the activation of the integrin-β1/FAK pathway by mechanical stress: abnormal mechanical stress activates FAK through integrin-β1 cell adhesion signals, and further phosphorylates P53 to enhance its inhibitory effect on SLC7A11 transcription; on the other hand, it can activate NOX1 to produce ROS, accelerating the ferroptosis process. In the DMM model, abnormal mechanical stress caused by joint instability can significantly increase iron accumulation and the expression of ferroptosis markers in cartilage tissue, while the use of ferroptosis inhibitors can effectively alleviate mechanical stress-induced cartilage degeneration, suggesting that mechanical stress participates in the OA pathological process by regulating chondrocyte ferroptosis.

In temporomandibular joint OA (TMJ OA) shown in **Figure 6**, such abnormal mechanical stress (MF) is also a core inducing factor—when the temporomandibular joint is subjected to excessive mechanical load for a long time, it will promote BRD4 to bind to the promoter region of Trem1 to enhance its expression, thereby activating molecular pathways such as the MAPK pathway and TRP channels (49, 50). On the one hand, it upregulates the release of inflammatory factors such as IL-1β and IL-6, as shown by the increased inflammatory signals in the cytoplasm in **Figure 6**; on the other hand, these inflammatory signals can reversely aggravate the abnormal expression of ferroptosis-related molecules in chondrocytes, ultimately jointly promoting the pathological changes of TMJ OA such as cartilage thinning and subchondral bone resorption shown in the figure; **Figure 6** also shows that after inhibiting BRD4 with JQ1 or siBRD4, the expression of Trem1 is downregulated, inflammatory signals are weakened, and these pathological damages are alleviated, which further indicates that mechanical stress can play a key driving role in the OA process of different joints through the synergistic network of “mechanical stimulation-inflammatory pathway-ferroptosis”.

### 4.2 Hypoxic microenvironment and chondrocyte ferroptosis

The hypoxic microenvironment of articular cartilage plays a central role in promoting ferroptosis in chondrocytes. Under inflammatory conditions or mechanical stress, hypoxia-inducible factor-1α (HIF-1α) is activated, driving the upregulation of transferrin receptor 1 and iron uptake, while simultaneously suppressing antioxidant defenses such as SLC7A11 and GPX4 (14). This imbalance promotes mitochondrial dysfunction, lipid peroxidation, and reactive oxygen species accumulation, culminating in ferroptotic cell death. In temporomandibular joint and knee OA models, interventions that inhibit HIF-1α or block iron import effectively reduce ferroptosis and preserve cartilage integrity (51), highlighting the pivotal role of hypoxia signaling in regulating chondrocyte susceptibility to ferroptosis.

Beyond classical hypoxia pathways, recent studies demonstrate that metabolic and epigenetic regulators intersect with hypoxia-driven ferroptosis. For example, SDF-1-mediated IL6/HIF-1α signaling establishes a positive feedback loop that amplifies iron overload and oxidative stress, while LDHB-mediated histone lactylation enhances ACSL4 expression, further sensitizing chondrocytes to lipid peroxidation (52, 53). These findings suggest that the hypoxic microenvironment not only directly triggers ferroptosis but also integrates inflammatory, metabolic, and epigenetic cues to exacerbate OA progression, offering multiple potential therapeutic targets within this niche.

### 4.3 Mutual influence between synovitis and chondrocyte ferroptosis

Synovitis is also one of the important pathological features of OA. Inflammatory cytokines released by immune cells such as synovial fibroblasts (FLS) and macrophages can stimulate chondrocyte ferroptosis through paracrine, and DAMPs released by chondrocyte ferroptosis can further aggravate synovitis, forming a “inflammation-ferroptosis” vicious circle.

After being stimulated by IL-1β or TNF-α, FLS and macrophages in OA synovial tissue are activated and release a large amount of ROS and pro-inflammatory factors, which can diffuse into cartilage tissue through the synovial-cartilage interface and act on chondrocyte surface receptors (22, 54), thereby activating the NF-κB and P38 MAPK signaling pathways, upregulating the expression of TfR1 and ACSL4, and inhibiting the expression of SLC7A11 and GPX4, inducing chondrocyte ferroptosis. At the same time, DAMPs such as HMGB1 and mitochondrial DNA released during chondrocyte ferroptosis are recognized by pattern recognition receptors (TLR4) on the surface of synovial cells, activating synovial cells to produce more inflammatory factors and ROS, aggravating synovitis and chondrocyte ferroptosis.

Evidence shows that targeting synovitis can significantly inhibit chondrocyte ferroptosis: administration of TNF-α neutralizing antibodies or NF-κB inhibitors in OA models reduces synovitis, significantly downregulates the expression of ferroptosis markers in cartilage tissue, and reverses cartilage degeneration (55, 56); inhibiting chondrocyte ferroptosis can reduce the release of DAMPs and alleviate synovitis, suggesting that breaking the “synovitis-chondrocyte ferroptosis” vicious circle may provide an effective method for the treatment of OA.

**Figure 7:**
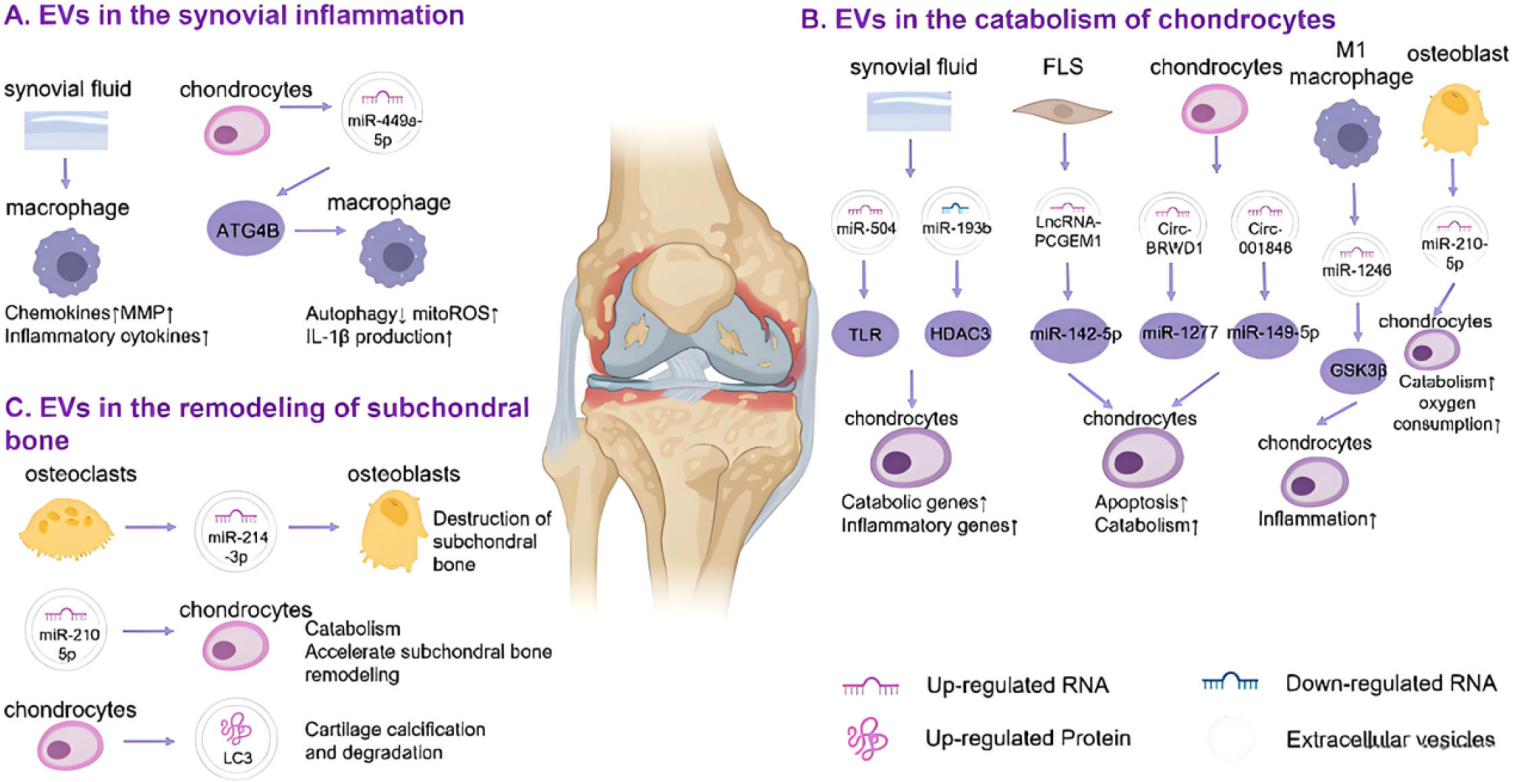
Synovitis and chondrocyte ferroptosis. **(A)** EVs from synovial fluid (SF) and chondrocytes affect macrophages, leading to increased synovitis. **(B)** EVs from the joint microenvironment affect chondrocyte metabolism, resulting in increased catabolism and cartilage matrix destruction. **(C)** EVs from osteoclasts lead to abnormal remodeling of subchondral bone.

The extracellular vesicles (EVs) shown in **Figure 7** are the key signal transmission carriers of this “synovitis-chondrocyte ferroptosis” vicious circle—FLS, macrophages, and chondrocytes activated by synovitis will release EVs carrying specific miRNAs (miR-448-5p, miR-193b), lncRNAs (lncRNA-PGEM1), or proteins, as shown in **Figures 7A, B**, which transmit transcellularly between synovium-cartilage-subchondral bone (57, 58): miR-448-5p carried by chondrocyte EVs in A can enhance macrophage autophagy and mitochondrial ROS production, further promoting the release of pro-inflammatory factors such as IL-1β to aggravate synovitis; EVs from FLS or M1 macrophages in B transmit regulatory molecules to chondrocytes, both promoting their catabolism and exacerbating their ferroptosis by affecting ferroptosis-related pathways; at the same time, EVs from chondrocytes or osteoclasts in C can also promote subchondral bone destruction and cartilage calcification, reversely worsening the joint microenvironment. This makes the vicious circle further amplified with the help of EVs, and also suggests that targeting the contents or release process of EVs may become a new direction for OA intervention.

## 5 Research progress on osteoarthritis therapeutic strategies targeting chondrocyte ferroptosis

### 5.1 Iron chelators

Iron chelators inhibit ferroptosis by specifically binding intracellular free Fe²^+^, reducing iron overload and the Fenton reaction, which is a classic therapeutic strategy targeting ferroptosis. Currently, iron chelators used in OA research mainly include deferoxamine (DFO), deferiprone (DFP), and deferasirox (DEF) (59, 60).

DFO is a classic iron chelator. In OA cell models, it can reduce the content of Fe²^+^, thereby reducing the production of ROS and lipid peroxides, and increasing the expression of GPX4 to improve chondrocyte activity and promote ECM synthesis (PubMed). *In vivo* studies have found that intra-articular injection of DFO significantly reduces cartilage destruction in DMM mice, downregulates Mankin score and OARSI score, and reduces the expression of synovial inflammatory factors (PubMed). DEF is an oral iron chelator with high bioavailability. A recent study linked DEF and PTE via a thioketal bond and loaded Ce to prepare ROS-responsive nanoparticles (61, 62). This nanodrug can release drugs under the condition of high ROS in the OA inflammatory microenvironment, and exert iron chelating, antioxidant, and anti-inflammatory effects. It can significantly improve chondrocyte ferroptosis *in vitro* and joint function in OA model mice *in vivo* without causing obvious organ toxicity, showing good clinical transformation prospects.

However, traditional iron chelators are limited in clinical use due to poor tissue targeting and short half-life. The development of new targeted delivery systems (such as chondrocyte-specific antibody-modified liposomes, hyaluronic acid-modified nanoparticles) can greatly increase the enrichment of iron chelators in cartilage and reduce systemic toxic side effects, bringing hope for the clinical transformation of iron chelators (63, 64).

### 5.2 Ferroptosis inhibitors

Ferroptosis inhibitors directly act on key links of the ferroptosis pathway, inhibiting lipid peroxidation or activating the antioxidant system, mainly including GPX4 activators and lipid peroxidation inhibitors.

Fer-1 is the most commonly used ferroptosis inhibitor, which can inhibit ferroptosis by directly scavenging lipid peroxyl radicals and has been widely verified in OA. *In vitro* experiments have found that Fer-1 can reverse chondrocyte ferroptosis induced by Erastin or IL-1β, restore the expression of COL2A1 and proteoglycans, and inhibit the secretion of MMP13. *In vivo* experiments have proved that oral or intra-articular injection of Fer-1 can significantly alleviate cartilage degeneration and joint pain symptoms in OA model animals (47, 65), improve joint motor ability, and have good long-term safety (PubMed). Another potent ferroptosis inhibitor, Liproxstatin-1, has a similar mechanism of action to Fer-1 and has obvious cartilage protective effects in OA models, but it is mainly used in basic research due to poor water solubility (PubMed).

In addition to classic inhibitors, there are new advances in GPX4 activators. For example, MK8722 specifically binds to the Sesn2 protein to activate the Sesn2-BNIP3-Nrf2 pathway, stimulating mitophagy and the expression of GPX4, and inhibiting chondrocyte ferroptosis; *in vitro* experiments have also confirmed that MK8722 significantly reduces the levels of ROS and MDA in chondrocytes stimulated by IL-1β and upregulates the expression of COL2A1; *in vivo* experiments have shown that oral administration of MK8722 can significantly improve cartilage degeneration and osteophyte formation in DMM model mice (66, 67), and the effective dose has no obvious organ toxicity, showing good therapeutic prospects.

### 5.3 Natural products and active components of traditional Chinese medicine

Natural products and active components of traditional Chinese medicine have attracted much attention in OA treatment due to their multi-target and low toxicity characteristics. Some studies have confirmed that they can exert therapeutic effects by inhibiting chondrocyte ferroptosis.

Irisin is a myokine induced by exercise. Recent studies have found that it can alleviate OA by regulating ferroptosis-related genes. In cell experiments, irisin can significantly reverse chondrocyte ferroptosis induced by TNF-α and Erastin, increase GSH content and mitochondrial membrane potential, and reduce MDA, ROS, and Fe²^+^ levels; mechanistically, irisin can upregulate the expression of GPX4 and SLC7A11 (67, 68), downregulate the expression of ferroptosis-promoting genes such as HMOX1, G6PD, and ALOX5 by inhibiting the ERK signaling pathway. HMOX1, G6PD, and ALOX5 have been proven to be key genes for OA ferroptosis (AUC > 0.9).

### 5.4 Nanodrugs and targeted delivery systems

Nanodrugs have the advantages of strong targeting, high drug loading efficiency, and microenvironment-responsive release, providing a new technical platform for OA treatment targeting chondrocyte ferroptosis. Current research mainly focuses on ROS-responsive, pH-responsive, and receptor-mediated targeted nano-delivery systems.

Ce@D&P NPs are recently designed ROS-responsive nanodrugs composed of DEF, PTE, and Ce³^+^ linked by thioketal bonds, which break the thioketal bonds to release drugs under the condition of high ROS in the OA inflammatory microenvironment. DEF acts as an iron chelator, PTE as an antioxidant and anti-inflammatory agent, and Ce³^+^ can mimic SOD and CAT activities to eliminate ROS (69, 70). In summary, the combined use of the three can significantly reduce the occurrence of chondrocyte ferroptosis, and inhibit synovitis and ECM degradation. *In vivo* studies have shown that intra-articular injection of Ce@D&P NPs can significantly improve the histological score of cartilage in DMM mouse models, reduce osteophyte formation, and cause no pathological damage to major organs such as the liver and kidneys, showing good biocompatibility and therapeutic effects.

In addition, HA-modified liposomes, as a cartilage-targeted delivery carrier, can increase the accumulation of drugs in cartilage by utilizing the specific recognition and binding between HA and the receptor CD44 on chondrocytes. Researchers have encapsulated Fer-1 in HA-modified liposomes. After intra-articular administration, it can effectively enhance the accumulation of Fer-1 in cartilage, extend its half-life, and its efficacy is significantly higher than that of free Fer-1, with reduced systemic toxicity (53, 71). The above studies have proved that nanodrugs and targeted drug delivery carriers can greatly enhance the effect of ferroptosis-related drugs, providing a new idea for the targeted treatment of OA.

## 6 Research controversies and future directions

Although the role of chondrocyte ferroptosis in OA has been widely confirmed, there are still some research controversies, mainly focusing on the following aspects:

There is a chronological issue regarding whether ferroptosis is the initiating factor of OA occurrence and development. On the one hand, some scholars propose that ferroptosis is an early initiating event of OA, and found that changes in ferroptosis-related indicators occur in the early stage of cartilage degeneration and the application of ferroptosis inhibitors can slow down OA progression (72, 73); on the other hand, some scholars point out that ferroptosis may occur in the late stage of OA, which may be a result rather than a cause of cartilage degeneration, mainly playing a role in accelerating disease progression rather than initiating pathological processes (PubMed). This contradiction may be related to the OA model used, the time points tested, and the type of samples, which still needs to be confirmed by prospective clinical observations and dynamic animal experiments.

The role of some regulatory factors is controversial. For example, there are conflicting reports on the impact of the NF-κB pathway on chondrocyte ferroptosis: most studies confirm that activating NF-κB can promote ferroptosis; however, a small number of studies have found that the use of NF-κB inhibitors has no obvious inhibitory effect on ferroptosis in some OA models, and it can aggravate iron overload by downregulating the expression of Ferritin protein. This difference may be related to the different subtypes of the NF-κB signaling pathway, different activation levels, and differences in the cellular microenvironment. Further research is needed on the complexity of its regulatory process (74, 75).

Based on the current research status and controversies, future research on chondrocyte ferroptosis in OA should focus on the following directions: Further clarify the cell-specific regulatory mechanism of ferroptosis. Through single-cell transcriptome, spatial transcriptome and other technologies, clarify the differences in ferroptosis sensitivity and regulatory mechanisms of different subpopulations of chondrocytes (such as proliferative chondrocytes, hypertrophic chondrocytes) in OA cartilage tissue, identify specific molecular markers of ferroptosis, and provide new targets for the early diagnosis of OA (PubMed). Elucidate the network regulation between ferroptosis and other OA pathological processes. Further explore the relationship between ferroptosis and pathological processes such as chondrocyte hypertrophy and differentiation, ECM degradation, and pannus formation, determine the key regulatory nodes in the “ferroptosis-inflammation-metabolic disorder” network, and provide a theoretical basis for multi-target intervention. Promote clinical transformation research targeting ferroptosis. Develop new drugs with high cartilage targeting, long half-life, and good biocompatibility (such as nanodrugs, small molecule compounds), and conduct preclinical safety and efficacy evaluations; design prospective clinical studies to verify the value of ferroptosis-related markers in OA diagnosis and prognosis assessment, and carry out research on the effect of ferroptosis-targeted drugs on OA (76). The complex pathological mechanism of OA determines that a single target targeting ferroptosis may not achieve good therapeutic effect. In future research, the combination of ferroptosis inhibitors with other drugs such as anti-inflammatory drugs, ECM protectants, and cartilage repair agents should be sought to exert a synergistic effect to improve the therapeutic effect, providing a new idea for the clinical application of OA.

## 7 Conclusion

OA is a prevalent degenerative joint disease characterized by complex interactions between chondrocyte death, extracellular matrix degradation, and pathological microenvironmental factors. Beyond apoptosis and autophagy, ferroptosis has emerged as a novel form of programmed cell death that contributes to OA progression through dysregulated iron metabolism, lipid peroxidation, and collapse of the antioxidant defense system. Chondrocyte ferroptosis interacts with inflammatory signaling, mechanical stress, and mitochondrial dysfunction, amplifying cartilage damage and disease progression.

Despite significant preclinical evidence demonstrating the potential of ferroptosis-targeted interventions—such as iron chelators, ferroptosis inhibitors, and nanodrug delivery systems—several critical questions remain. Future research should address the diagnostic and prognostic value of ferroptosis-related biomarkers, clarify joint- and cell type-specific regulatory mechanisms, explore the interplay between ferroptosis and other forms of cell death, and evaluate combination therapies that integrate ferroptosis modulation with anti-inflammatory or cartilage-protective strategies. Advancing these areas will be essential for translating mechanistic insights into safe and effective clinical interventions for OA patients.
